# Personalized and Precision Medicine in Asthma and Eosinophilic Esophagitis: The Role of T2 Target Therapy

**DOI:** 10.3390/pharmaceutics15092359

**Published:** 2023-09-21

**Authors:** Diego Bagnasco, Edoardo Vincenzo Savarino, Mona-Rita Yacoub, Fulvio Braido, Maria Giulia Candeliere, Edoardo Giannini, Giovanni Passalacqua, Elisa Marabotto

**Affiliations:** 1 Allergy and Respiratory Diseases, IRCCS Policlinic San Martino, University of Genoa, 16132 Genoa, Italy; 2 Department of Internal Medicine (DIMI), University of Genoa, 16132 Genoa, Italy; 3 Department of Surgical Oncological and Gastroenterological Sciences, University Hospital of Padova, 35145 Padua, Italy; 4 Unit of Immunology, Rheumatology, Allergy and Rare Diseases, IRCCS Hospital San Raffaele, 20132 Milan, Italy; 5 Faculty of Medicine, Vita-Salute San Raffaele University, 20132 Milan, Italy; 6 Gastroenterology Unit, Department of Internal Medicine, IRCCS Ospedale Policlinico San Martino, University of Genova, 16132 Genova, Italy

**Keywords:** severe asthma, eosinophils, eosinophilic esophagitis, biologics, T2 inflammation, allarmins, TSLP, cytokines

## Abstract

The role of type 2 inflammation has been progressively associated with many diseases, including severe asthma, atopic dermatitis, nasal polyposis, eosinophilic granulomatosis with polyangiitis, and, recently, eosinophilic esophagitis. Despite this, the association between asthma and esophagitis is still poorly known, and this is probably because of the low prevalence of each disease and the even lower association between them. Nonetheless, observations in clinical trials and, subsequently, in real life, have allowed researchers to observe how drugs acting on type 2 inflammation, initially developed and marketed for severe asthma, could be effective also in treating eosinophilic esophagitis. For this reason, clinical trials specifically designed for the use of drugs targeted to type 2 inflammation were also developed for eosinophilic esophagitis. The results of clinical trials are presently promising and envisage the use of biologicals that are also likely to be employed in the field of gastroenterology in the near future. This review focuses on the use of biologicals for type 2 inflammation in cases of combined severe asthma and eosinophilic esophagitis.

## 1. Introduction

Type 2 (T2) inflammation was identified as a pathogenic phenomenon underlying various diseases, such as asthma, nasal polyposis, and atopic dermatitis. The eosinophilic inflammatory infiltrate, which is typical of T2 inflammation, has been demonstrated to be present in other diseases, including eosinophilic esophagitis (EoE). Asthma is a well-known disease, characterized by chronic airway inflammation and bronchial hyperresponsiveness, resulting in airflow limitation and respiratory symptoms. In the pathogenesis of asthma, interactions between genetic predisposition, environmental factors, and immune dysregulation play a role in the genesis of disease. The hallmark of asthma is chronic airway inflammation, primarily driven by type 2 (T2) immune responses. This immune activation results in the recruitment of eosinophils, mast cells, IgE, and other inflammatory cells to the airways, leading to structural changes and remodeling, increased mucus production, and bronchospasm. Several cytokines, including interleukin (IL)-4, IL-5, IL-13, IL-25, and IL-33, thymic stromal lymphopoietin (TSLP), and cells such as innate lymphoid cells type 2 (ILC2s), play crucial roles in orchestrating the inflammatory response in asthma. Among the cells responsible for inflammation that are regulated by the inflammatory pathway related to the cytokines earlier mentioned, are eosinophils. These cells are increased in both the blood and airway tissues of asthma patients and have become direct or indirect targets of many drugs developed to be controllers of asthma. With the development of knowledge regarding the pathophysiological mechanisms of T2-defined inflammation, it has been possible to associate some diseases with asthma, not only epidemiologically, but also using the inflammation pathway. These certainly include chronic rhinosinusitis with nasal polyposis (CRSwNP) and atopic dermatitis, which have a rather high incidence of coexistence with asthma. In contrast, diseases that are less frequently associated with asthma include eosinophilic esophagitis (EoE), despite it being proven that the presence of type 2 inflammatory diseases significantly increases the risk of EoE [1]. It is estimated that patients with EoE are commonly affected by asthma in about 45.4% of cases [2]. On the other hand, a prevalence of 16.5% of EoE has been reported in allergic patients [3].

EoE is an emerging chronic immune-mediated disorder that primarily affects the esophagus, leading to esophageal dysfunction and associated symptoms. EoE is characterized by eosinophilic infiltration and inflammation of the esophagus. Patients with EoE often present with symptoms such as dysphagia, food impaction, chest pain, and heartburn, mimicking gastroesophageal reflux disease (GERD). However, unlike GERD, EoE usually does not respond to acid-suppressive therapy. As previously described in asthma, the pathogenesis of EoE also involves a combination of genetic predisposition, environmental triggers, and dysregulated immune responses. Similar to asthma, the T2 immune response is also a key driver of the inflammatory process in EoE. Eosinophils and other inflammatory cells infiltrate the esophageal tissue, leading to mucosal damage, fibrosis, and impaired esophageal motility. Cytokines such as IL-4, IL-5, and IL-13, as well as eotaxins—chemokines involved in eosinophil recruitment—contribute to the pathogenesis of EoE. Although both conditions involve inflammation and share some similarities, they affect different anatomical sites and exhibit distinct clinical manifestations.

Despite the evident common inflammatory pathway, there are not many studies linking these two diseases. About 10 years ago, Virchow defined EoE as “*asthma of the esophagus*” due to the similarities between diseases to emphasize the similarities of two diseases too often regarded as two entities that are distinctly separate from each other [4]. 

Understanding the shared and distinct mechanisms of inflammation in asthma and EoE is essential for developing targeted therapies. Current treatments for both conditions focus on controlling symptoms and reducing inflammation. In asthma, inhaled corticosteroids, long-acting beta-agonists, leukotriene modifiers, and monoclonal antibodies targeting specific cytokines have revolutionized management. Similarly, in EoE, dietary management, proton pump inhibitors, and topical corticosteroids are the mainstay of current treatments. However, there is still an unmet need for more effective and personalized therapies that can address the underlying inflammatory processes and provide long-term remission.

The development of biological drugs (specifically, those directed toward components of T2 inflammation) and several sub-analyses of clinical trials have made it possible to evaluate the effect of biologicals initially developed for asthma on eosinophilic esophagitis. The results obtained so far are encouraging and contribute to the knowledge of this type of inflammation; moreover, they provide useful suggestions when both diseases are present to orient a clinician’s therapeutic choices. 

The research of both pathologies is crucial, both to know the real prevalence of asthma in EoE patients and vice versa and to confirm that the same drug can affect both pathologies, allowing the clinician to choose one molecule, rather than another, in the case of comorbid patients.

This manuscript provides an overview of the inflammatory mechanisms underlying asthma and eosinophilic esophagitis, highlighting their etiology, pathogenesis, and potential therapeutic strategies.

## 2. Type 2 Inflammation

**Figure 1:**
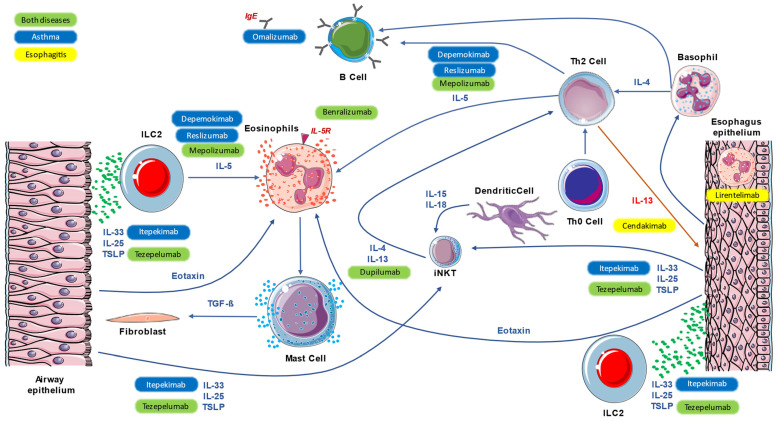
Main mechanisms of common inflammation, both in asthma and eosinophilic esophagitis, and the action of monoclonal antibodies.

The type 2 immune response encompasses both the innate and adaptive arms of the immune system and represents the common inflammatory pathway within a broad range of inflammatory and infectious diseases [5]. Historically, atopic diseases have mainly been the paradigm of the T2 immune response; however, in recent years, insights into the pathophysiology of other eosinophilic inflammatory diseases have expanded the spectrum of diseases characterized by type 2-driven inflammation [6]. Accordingly, scientific efforts are currently being made to translate the immunological knowledge from the “classic” diseases studied for decades (i.e., asthma, CRSwNP, and atopic dermatitis) to other emerging diseases such as eosinophilic esophagitis. Besides the characteristic tissue eosinophilic inflammation, one main characteristic is the high prevalence of atopy in these patients. Atopy certainly contributes to the epithelial barrier dysfunction that characterizes these conditions; however, multiple other factors can intervene, such as genetic predisposition and epigenetics, both external (protease, irritants, particulate matter, injury, and viruses) and internal (hormones, growth factors, diet, and changes in the commensal microbiome). Type 2 immune-mediated inflammation is orchestrated by a complex interaction between Th2 cells and group 2 innate lymphoid cells (ILC2), which produce type 2 cytokines, including interleukin IL-4, IL-5, and IL-13, as well as other inflammatory mediators [7,8]. Th2 cells are a subset of T helper CD4 that are characterized by the production of Th2 cytokines. They are often observed in the tissue of allergic patients but are also involved in the pathogenesis of other inflammatory diseases and the defense against helminthic infection. Pathogenic Th2 cells are a subgroup of Th2 that are capable of producing high amounts of IL-5 and IL-13 in response to epithelial cell damage, which leads to an amplification of eosinophilic tissue inflammation. Tpath2 also plays an important role in fibrogenesis, particularly through amphiregulin, a member of the epidermal growth factor family produced by epithelial cells undergoing tissue injury [9]. IL-4 upregulates Th2 cells and promotes the differentiation of B lymphocytes into plasma cells, producing IgE. IL-5 plays a central role in stimulating the differentiation and maturation of eosinophil progenitors in bone marrow, as well as their trafficking and survival [10,11]. IL-13 is a pivotal regulator of IgE synthesis, mucus hypersecretion, and fibrosis. It promotes eosinophils survival, activation, and recruitment; IL-13 is also related to epithelial barrier disruption via the downregulation of epithelial junction molecules [12]. Innate lymphoid cells type 2 (ILC2) are the innate counterpart of the adaptive Th2 cells [13]. These lymphocyte-like cells were recently identified as a major component of mucosal immunity, as they are mainly activated by epithelium-derived cytokines, including the alarmins IL-33, IL-25, and thymic stromal lymphopoietin (TSLP). Indeed, ILC2 cells are also a main source of type 2 cytokines during allergic inflammation and helminth infection. ILC2 cells also produce IL-9, which promotes self-survival and, therefore, amplifies type 2 cytokine production [14,15]. IL-25, IL-33, and TSLP are epithelial cytokines, also known as alarmins, that are released by epithelial cells in response to allergic and non-allergic triggers such as viruses, cigarette smoke, and pollution. IL-9 upregulates the expression of IL-5 and IL-13 by ILC2 in positive autocrine feedback and promotes mast cell survival and activation [16]. The crosstalk between innate ILC2 and Th2 cells leads, under certain conditions, to T2-high inflammatory diseases, such as atopic dermatitis, asthma, allergic rhinitis, CRSwNP, or eosinophilic esophagitis. The pathogenesis of these conditions can follow the well-known allergic pathways (IgE-mediated mast cells and basophils degranulation); however, more often, more complex immune-mediated mechanisms are derived from the interactions between the genetic background and the environmental exposure [17]. Type 2 inflammation mechanisms, which are common in asthma and esophagitis, are summarized in Figure 1.

## 3. T2 Targeted Therapies in Asthma

The identification of the phenotype first [18], and the endotype later [19,20], in asthmatic subjects, is crucial when choosing the more appropriate therapy (Table 1). The GINA document suggests that, after adequately checking for the correct diagnosis, control of comorbidities, and monitoring adherence to prescribed inhaled therapy, an attempt to endotype patients according to certain markers related to T2 inflammation should be made [21]. The characteristics of this inflammation have been previously described. Specifically, in clinical practice, eosinophils, exhaled nitric oxide, allergy, or an eosinophil count in sputum greater than 2% are currently used as markers [21]. The first therapeutic target in asthma in patients with T2 inflammation was, for historical reasons, IgE [22], which was initially antagonized with the monoclonal antibody omalizumab [23,24]. Subsequent targets were IL-5 [25], with the direct cytokine antagonist mepolizumab [26,27,28,29,30,31] and reslizumab [32,33,34,35], IL-5 receptor (IL-5r) with benralizumab [36,37,38,39,40], and IL-4 receptor (IL-4r) with dupilumab [41,42,43]. To both these targets and drugs, tezepelumab [44,45], an antibody directed at TSLP [46,47,48,49,50,51], is currently being marketed. 

In cases of severe asthma, the use of several biological drugs has achieved a significant reduction in exacerbations, the need for systemic corticosteroid use, and, in some cases, an improvement in respiratory function [24,52,53]. The aforementioned goals are the main parameters, which were evaluated in clinical trials to assess the effect of biologics on severely asthmatic patients. The first results were obtained in the early 2000s using omalizumab in allergic asthma, showing a reduction in exacerbations in patients treated with the drug. Despite other marketed therapies using eosinophils as predictive markers of efficacy, none were observed for omalizumab, and trials have demonstrated that the efficacy of the drug was found regardless of the presence or not of these cells [54]. Furthermore, regarding observations on the safety of the drug, data on reports related to carcinogenesis were analyzed, leading to the conclusion that the drug is safe and does not increase neoplastic risk [55].

Subsequently, other biological drugs targeting IL-5 were developed, namely, mepolizumab and reslizumab. Regarding the former, data from clinical and real-life trials showed its efficacy in reducing disease exacerbations in patients with an eosinophil count above 150 cells/mcl, allowing, in addition, a significant reduction in daily dosing and, in most cases, the discontinuation of systemic steroid therapy [29,56]. Other real-life data demonstrated that the efficacy of mepolizumab is independent of the blood eosinophil count, as long as they comprise at least 150 cells/mcl [31,57]. Similar evidence came from reslizumab, although a smaller amount of data is available since the marketing of this drug has not been commercialized in all countries [58].

Real-life data has also suggested that mepolizumab is equally effective in patients with and without certain comorbidities such as nasal polyposis [59,60,61,62,63,64,65], also indicating that patients with this condition have a better response to the drug [66]. 

Again, observations in real life have shown the efficacy of the drug in other diseases such as eosinophilic esophagitis [67,68,69]. Mepolizumab was approved for two orphan diseases, namely, EGPA [70,71,72] and HES [73,74,75]. Concerning benralizumab, the effect on the reduction in exacerbations and the need for systemic corticosteroids was confirmed in randomized clinical trials [76]. Despite different mechanisms of action, this time directed against the alpha receptor of IL-5, benralizumab demonstrated its efficacy in severely asthmatic patients with similar characteristics to the one treated with anti IL-5 drugs. In this case, the effect of the drug on eosinophils is not caused by the modulation of cytokine, but through the intervention of natural killer (NK) cells that are able to send eosinophils in apoptosis and thereby inhibit their bronchial and tissue inflammatory action. In addition to its effectiveness, benralizumab also demonstrated reassuring safety in patients treated in both real-life and clinical trials. 

As with mepolizumab, although there is no primary indication for this condition, in real life, it has been shown to be effective on nasal polyposis, and this comorbidity has again been associated with improved therapeutic response in patients treated for asthma [40,77,78,79,80,81]. 

Similarly, against IL-5, reslizumab was able to reduce asthma exacerbations and OCS intake in treated patients; unlike other drugs, it was administered intravenously, which is a method that is yet to be approved in several countries [32,33,34,35,82]. As for other drugs, reslizumab was evaluated both in naïve patients and in the switcher group, ensuring results in both groups [83]. 

Dupilumab, another drug directed on T2 inflammation mechanisms, was precisely directed to the IL-4 receptor, which is a molecule that can interact both with IL-4 and IL-13 (currently used in atopic dermatitis, nasal polyposis, and asthma). The administration of dupilumab has been demonstrated to be able to reduce exacerbations and systemic corticosteroid use, and, regarding nasal polyposis, it showed efficacy in reducing the polyp volume [81,84,85,86]. Dupilumab, in addition to what has been described above, is very effective in improving respiratory function in treated patients, providing an important result regarding disease control. The more ubiquitous mechanism of action of dupilumab also allows it to interact, albeit indirectly, with the action of IgE and, thus, on some of those mechanisms that may be most characteristic of patients with an allergic form of asthma, allowing clinicians to consider it for this type of patient as well as for their therapy [83].

Lastly, tezepelumab, an anti-TSLP antibody, is the first drug to interfere with the alarmin chain. One of the most interesting aspects of the molecule is precisely its action on a chain of inflammation beyond that of specifically type 2 cytokines (IL-5, IL4, IL-13), acting on what is produced as a result of epithelial damage.

Current available data demonstrate a significant efficacy in patients with T2 inflammation, as well as partial efficacy in those with T2-low characteristics [44,45,87,88,89,90,91,92]. Clinical trial data show the efficacy of tezepelumab, principally in patients with high levels of eosinophils; however, for the first time in severe asthmatic therapies, interesting results have been obtained on patients with less than 150 eosinophils/mcl, despite having a slightly lower efficacy score, if compared to the sample with a range higher than the above-mentioned eosinophils range. Stimulating results appear also in the trial regarding the effect of the molecule on bronchial hyperactivity (AHR) and local inflammation, demonstrating that the reduction in eosinophilic inflammation was greater in patients treated with tezepelumab than in the one randomized in the placebo arm, from which analog results were obtained regarding AHR. 

The results about hyperactivity include differentiators from other drugs available for asthma at this time; in fact, omalizumab was able to reduce AHR only in several studies using methacholine, acetylcholine, or adenosine monophosphate; mepolizumab did not achieve this result as no results were obtained with benralizumab, lebrikizumab, tocilizumab, efalizumab, and tralokinumab. The only other drug, which is currently not marketed for asthma, that achieved the result of reducing AHR was etanercept in one study. In the end, the only two drugs that are currently available for asthma and able to reduce AHR are tezepelumab and omalizumab [93]. 

Regarding efficacy on OCS-dependent patients, however, in clinical trials, tezepelumab did not meet the endpoint of the reduction in exacerbations in this category of patients. Sub-analyses of the dedicated study, however, showed that the administration of the biologic drug was able reduce exacerbations and OCS use in dependent patients, with results being proportionally related to the eosinophilic count at baseline. A new clinical trial with this aim is ongoing [94]. The long-term effects on tezepelumab safety were evaluated in the Navigator trial, a 104-week extension of short-term trials, and no difference between the active and placebo arm groups was described. 

Currently, a new anti IL-5 biological, depemokimab, is under investigation. Its distinguishing characteristic is its long-life capability, which allows for administration every 6 months [95]. 

Two molecules targeting IL-13 have been under study for several years, with controversial results. Certainly, the addition of the effect on IL-4r and not only IL-13 demonstrates a more effective result. Lebrikizumab (anti IL-13) was demonstrated to be effective on adolescents (12–17 years) in reducing the exacerbation rate after 52 weeks, despite the fact that the study was prematurely terminated (sponsor’s decision), potentially limiting our interpretation of the results [96]. Lebrikizumab was also able to reduce baseline blood eosinophilia or FeNO, which were usually used as biomarkers in the Lavolta study [97]. Regarding other therapies directed at IL-13, the results of clinical trials on the use of tralokinumab show poor efficacy in terms of exacerbation reduction; however, in another instance, as previously described for lebrikizumab, a reduction in biomarker levels could be described [98]. As for exacerbations, the OCS-sparing effect was also not achieved in trials [99]. 

Other therapeutic targets under study, at this time, are being directed toward mediators of type 2 inflammation, as well as TSLP, with CSJ117 [100], and IL-33 with itepekimab [101], which are being studied in moderate–severe patients undergoing ICS-LABA therapy, in addition to etokimab (ANB020) and melrilimab (GSK3772847) against IL-33, both in first experimental phase [100], astegolimab [102] and anti ST-2 (ILC2 receptor for IL-33), which can reduce asthma exacerbations in phase 2 trials.

## 4. T2 Target Therapy in Eosinophilic Esophagitis

Eosinophilic esophagitis (EoE) is an emerging disease that is defined by symptoms of esophageal dysfunction and abnormal eosinophilic inflammation within the esophagus. The diagnosis is histological and depends on the number of eosinophils detected (at least 15 eosinophils per high-power field in the absence of other causes of esophageal eosinophilia) [103,104,105].

The mechanism of inflammation is Th2-mediated, and aeroallergens/food antigens are the most important triggers. The migration to the esophageal wall and the activation of eosinophils and mast cells promote the release of proinflammatory cytokines, such as interleukin (IL)-4, IL-5, IL-13, and transforming growth factor (TGF) β, causing epithelial barrier disruption, smooth muscle impairment, and tissue remodeling [106,107,108,109]. The chronic inflammation in EoE patients causes esophageal fibrous remodeling with strictures that affect the patient’s quality of life. Therefore, EoE requires treatments to induce and maintain histological remission to stop the progression from inflammation to esophageal strictures [110]. Topical corticosteroids (STCs), proton-pump inhibitors (PPIs), and dietary changes are recommended by current guidelines to obtain this aim [103,104]. 

Nowadays, several new targeted therapies that can arrest the inflammation cascade are being investigated for EoE. Some drugs are imported from bronchial asthma and atopic dermatitis since these diseases have the same mechanism of inflammation [110] (Table 2).

Dupilumab, a monoclonal antibody that inhibits the action of IL-4 and IL-13 by blocking the shared IL-4 receptor α subunit has been approved by the FDA for moderate–severe atopic dermatitis, asthma, and rhinosinusitis with nasal polyposis treatment. It suppresses most Th2 inflammatory biomarkers, representing an optimal target drug for Th2-mediated diseases; moreover, for this reason, it was also studied in the context of EoE [111]. In May 2022, dupilumab was also approved in the treatment of EoE in phase 2 and 3 trials [112]. In particular, in a phase 2 trial versus placebo, adult patients with active EoE after 12 weeks of weekly subcutaneous dupilumab injections showed a significant improvement in dysphagia, histologic, and endoscopic features, as well as esophageal function, with an acceptable safety profile. A significant reduction in eosinophil counts was obtained, with 83% of patients achieving less than 15 eosinophils per high-power field [110]. Considering the small and short duration of the study, a subsequent study was conducted to evaluate the long-term efficacy and safety of this drug in this class of patients. Preliminary data from a phase 3 study confirmed its effectiveness in adolescent and adult patients after 52 weeks of weekly treatment [113,114].

Another monoclonal antibody that acts on IL-13 is cendakimab (previously RPC4046). It inhibits binding at the IL-513 receptor and is administered weekly via subcutaneous injections. It demonstrated a histological and endoscopic improvement in a 16-week phase 2 study in adults with active EoE but not a clinical remission, although an improving trend was shown [115]. An endoscopic and histologic improvement continues with an increase in the duration of treatment, as shown in the data from the open-label, long-term extension (LTE), wherein the percent of clinical and histological responders grows progressively both in placebo–cendakimab patients (14% at LTE entry to 67% at LTE week 52) and in cendakimab–cendakimab patients (30% at LTE entry to 54% at LTE week 52 [116].

IL-5, which is produced by T helper 2 cells, is involved in the differentiation, maturation, and migration process from the bone marrow of eosinophils favoring their adhesion to the esophageal wall. Eosinophils are directly involved in tissue remodeling as shown in an experimental model [117]. Therefore, strategies to reduce esophageal eosinophilic inflammation in patients with EoE were evaluated as a possible therapeutic strategy. Mepolizumab is a fully humanized anti IL-5 monoclonal antibody. It is currently approved by the FDA for the treatment of severe asthma in patients from the age of 6, hypereosinophilic syndrome in patients that are 12 years and older, and chronic rhinosinusitis with nasal polyps and eosinophilic granulomatosis with polyangiitis. In a randomized placebo-controlled, double-blind trial, mepolizumab showed a significant reduction in esophageal eosinophilia but an inconsistent symptom improvement [118]. Benralizumab is another monoclonal antibody directed against the membrane-bound IL-5 receptor α chain present in eosinophils. It is currently approved by the FDA for the treatment of severe asthma in patients 12 years and older. A phase 3 trial of benralizumab in EoE was recently stopped (in October 2022) because it did not meet one of the two dual-primary endpoints: it did not achieve clinical remission in the study population; rather, only a statistically significant improvement in histological disease compared to placebo.

Lymphocyte-trafficking modulation is currently being explored as a possible target for therapy in refractory eosinophilic gastro-intestinal disorders including EoE. Vedolizumab, a monoclonal integrin α4β7 antibody, mainly blocks CD4+T lymphocytes from binding to MAd CAM1 on intestinal endothelial cells, ensuring gut-selective, anti-inflammatory activity; for this reason, it has been approved to treat inflammatory bowel disease. In addition, it also blocks αE/β7 integrin, which has been found in Th2 lymphocyte cells in EoE patients, and this effect could lead to hypotheses regarding its role in the context of EoE patients [119,120]. Vedolizumab and natalizumab, two recombinant humanized anti-α4-integrin antibodies, have provided some benefit to EoE patients, albeit only case reports have been reported [121,122,123,124].

Lirentelimab (or antolimab) is a monoclonal antibody to sialic acid-binding immunoglobulin-like lectin 8 (Siglec-8), a CD33 receptor located on the surface of eosinophils and mast cells. It induces eosinophilic apoptosis and inhibits mast cell activation. In a small clinical trial, histologic remission (≤6 eos/hpf) was achieved, but there was no statistical significance for the patient-reported symptomatic coprimary endpoints; however, the results of these trials have not yet been published [125]. 

The ENIGMA trial, a randomized, phase 2, placebo-controlled study aiming to assess the efficacy of the anti Siglec-8 antibody (AK002) in adult patients with eosinophilic gastritis and gastroenteritis, has recently demonstrated histologic remission in patients with concomitant EoE [126]. For this reason, lirentelimab has also been evaluated in the context of EoE (KRIPTOS trial), with unfortunately similar evidence (histologic remission without clinical benefit) [127].

Tezepelumab, a TSLP antagonist used for asthma and atopic dermatitis, has a mechanism of action that targets the top of the inflammatory cascade, and a recent clinical trial has started to evaluate its role in improving outcomes for EoE patients [128,129]. 

Moreover, chronic inflammation leads to progressive esophageal remodeling, and TGF-β plays a relevant role in this process. Losartan, an antigotensin-1 receptor antagonist approved to treat high blood pressure, has been proposed due to its ability to reduce the signaling of TGF-β as a therapy in EoE patients. An ongoing phase 2 trial with increasing doses of losartan is ongoing [130].

Finally, the JAK–STAT pathway has been identified as a potential target in the treatment of EoE. There are no studies on the JAK inhibitor tofacitinib in EoE patients, but it has been reported that it was able to reduce esophageal eosinophilic infiltration in a patient with EoE [131]. 

The long-term efficacy of these drugs will be determined by further studies, but they seem to be promising in modifying the course of the disease.

## 5. Unmet Needs

**Figure 2:**
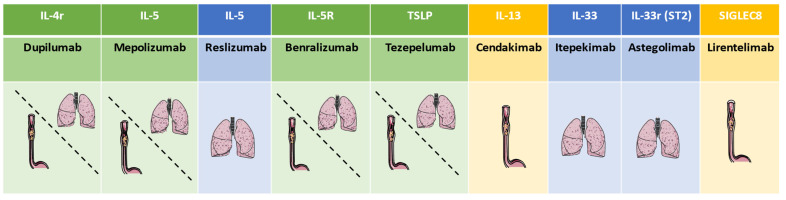
Principal antibodies and their use in asthma and esophagitis. (The green scale represents both diseases, blue scale represents only severe asthma, and yellow scale represents only esophagitis).

Although the effect of biologicals both in asthma and eosinophilic esophagitis was demonstrated, some points remain unclear and require further investigation (Figure 2). In the field of asthma, for example, the definition of the severity of a disease is not unique, and the classification criteria according to ATS/ERS [132], WHO [133], and NICE [134] differ. This means that a patient may or may not be diagnosed with severe asthma depending on the definition used [135]. This aspect could be overcome using the definition suggested by GINA in the last drafts, which almost completely overlaps with the ATS/ERS criteria [136]. The first point, which is common in asthma and esophagitis, concerns treatment adherence [137,138,139,140,141]. Currently, it is not possible to have clear and objective information on how many patients are taking the drugs prescribed to them, and this may inevitably lead to an increase in the prescription of biologic drugs to patients who are not truly severe, but only poorly adherent. Regarding asthma, some companies are studying add-on devices that can be connected to the inhaled medication to count the actual doses taken by the patient; however, the technology of these comma objects and their distribution still needs some time [142]. Regarding the treatment of esophagitis with drugs, developing this kind of technology for oral intake is still quite complicated. Another point in the field of severe asthma is the choice of biological drug, having several molecules with similar targets available. The choice of drugs currently falls on the presence or absence of markers such as eosinophilia, IgE, OCS dependence, and, empirically, by the simultaneous presence of comorbidities that can be attacked by the same molecule. In the field of esophagitis, a critical point that remains unresolved is the lack of real-life data, which are increasingly important in the management of a drug, as well as in understanding its potential. A further point of insight concerns the self-administration of biologic drugs that are now required to be increasingly produced in a way that makes home administration possible. With the patients’ ability to take the drug without physician supervision, adherence to this treatment may decline, thus becoming a problem from both a clinical and pharmacoeconomic perspective. At present, and for the foreseeable future, the common pathophysiological mechanisms of these two pathologies necessitate an investigation, that is at least in clinical history, of patients with respiratory comorbidities and esophagitis and patients with digestive comorbidities and asthma. In fact, the possibility of treating two diseases with a single drug makes it possible to carry out true precision medicine, targeting the pathophysiological process underlying both diseases.

## 6. Conclusions

In conclusion, the effect of eosinophils and T2 inflammation has been proven to be present both in asthma and eosinophilic esophagitis. In the field of asthma, the role of monoclonal antibodies is pivotal in the treatment of the severe form of this disease, which is currently not responding adequately to traditional therapy, to reduce OCS use; the real-life analysis and sub-analysis of randomized trials of patients treated for asthma and their pathology have demonstrated the effect of antibodies also in esophagitis. The development of trials dedicated to esophagitis provides interesting results and different targets which are also directed to T2 cytokines and mediators. Further studies will be needed to evaluate the relationship between asthma and eosinophilic esophagitis, with a search for common inflammatory pathways and interactions between the diseases, as has already been evaluated in rhinosinusitis with nasal polyposis, to better understand the role of type 2 inflammation and the possible causes of the imbalance of inflammatory mechanisms.

The evidence of a common inflammatory pathway in these two diseases dictates increased attention to the management of these patients. As the search for polyposis comorbidity has become more routinary over time in asthmatic patients, any pathology related to a similar mechanism, but located in a different district than the airway, must be considered. This review aimed to focus on raising awareness of one of the diseases related to T2 inflammation, that is generally seldom considered as a possible comorbidity of asthma, aiming to inform gastroenterologists and pulmonary-allergologists of the importance of the search for these two diseases.

## Figures and Tables

**Table 1:** Biologicals used and ongoing trials for severe asthma.

Molecule	Mechanism	Administration	Other Indications Approved
Benralizumab	Anti IL-5 receptor	s.c. 30 mg 4 weeks first 3 doses, 8 weeks other administrations	-
Dupilumab	Anti IL-4α receptor	400–600 mg s.c. first dose than 200–300 mg s.c. 2 weeks	Atopic dermatitis, nasal polyposis, prurigo nodularis, EoE *
Mepolizumab	Anti IL-5	s.c. 100 mg 4 weeks s.c. 300 mg 4 weeks in EGPA	Nasal polyposis, EGPA, hypereosinophilic syndrome
Reslizumab	Anti IL-5	e.v. 3 mg/Kg	Asthma
Omalizumab	Anti IgE	s.c. according to total IgE and weight	Nasal polyposis, chronic idiopathic urticaria
Tezepelumab	Anti TSLP	s.c. 210 mg 4 weeks	-
Clinical trials			
Astegolimab	Anti IL-33 receptor	-	-
CSJ117	Anti TSLP	-	-
Melrilimab	Anti IL-33	-	-
Etokimab	Anti IL-33	-	-
Itepekimab	Anti IL-33	-	-
Lebrikizumab	Anti IL-13	-	Atopic dermatitis
Tralokinumab	Anti IL-13	-	Atopic dermatitis

s.c. = subcutaneous; EGPA = eosinophilic granulomatosis with polyangiitis; * approved by EMA.

**Table 2:** Therapeutical approaches proposed for eosinophilic esophagitis.

Molecule	Mechanism	Administration	Phase of Trials
Benralizumab	Anti IL-5 receptor	30 mg SC every 4 weeks	Phase 3 trials recently stopped in October 2022
Cendakimab	Anti IL-13 receptor	180 or 360 mg once weekly for 16 weeks via subcutaneous injections	Phase 2 trial, long-term extension study
Dupilumab	Anti IL-4α receptor	300 mg subcutaneous weekly	Phase 3
Vedolizumab	Anti integrin α4β7/αE/β7	300 mg intravenously at 0, 2, and 6 weeks, then every 8 weeks	_
Natalizumab	Anti-α4-integrin	300 mg intravenously monthly	-
Lirentelimab	Monoclonal antibody to sialic acid-binding immunoglobulin-like lectin 8	Patients were randomized 1:1:1 to receive: 1.0 mg/kg of lirentelimab for the first month followed by five doses of 3.0 mg/kg given monthly (b) monthly 1.0 mg/kg of lirentelimab (c) a monthly placebo	Phase 2/3
Mepolizumab	Anti IL-5	s.c. 100/300 mg 4 weeks	Phase 2
Tezepelumab	Anti TSLP	subcutaneously using an accessorized pre-filled syringe	Phase 3
Losartan	Antigotensin-1 receptor antagonist	0.7–0.9 mg/kg (titration)/1.0–1.4 mg/kg (maintenance) daily for 16 weeks	Phase 2 trial

## Data Availability

No data were created.

## Abbreviations

CRSwNP	Chronic rhinosinusitis with nasal polyps
EGPA	Eosinophilic granulomatosis with polyangiitis
EOE	Eosinophilic esophagitis
IL	Interleukin
ILC2	Innate Lymphoid Cells Type 2
T2	Type 2
TSLP	Thymic stromal lymphopoietin
